# Biomarker Identification Using Text Mining

**DOI:** 10.1155/2012/135780

**Published:** 2012-11-11

**Authors:** Hui Li, Chunmei Liu

**Affiliations:** Department of Systems and Computer Science, Howard University, Washington, DC 20059, USA

## Abstract

Identifying molecular biomarkers has become one of the important tasks for scientists to assess the different phenotypic states of cells or organisms correlated to the genotypes of diseases from large-scale biological data. In this paper, we proposed a text-mining-based method to discover biomarkers from PubMed. First, we construct a database based on a dictionary, and then we used a finite state machine to identify the biomarkers. Our method of text mining provides a highly reliable approach to discover the biomarkers in the PubMed database.

## 1. Introduction 

Identifying molecular biomarkers has become an essential task for bioinformatics scientists to assess the different phenotypic states of cells or organisms correlated to the genotypes of diseases from large-scale biological data [1]. The text mining technique has become a critical technique for designing future predictive and personalized medicine. At the same time, the PubMed database which comprises more than 21 million citations for biomedical literature offers an enriched source for us to explore the biomarkers across human disease and to mine the biomarkers related to diseases. Therefore, integrating automatic literature searches, and text mining is a fast emerging research area in epigenetics, DNA methylation, and more specifically biomarker discovery studies. For almost every cancer type, new publications that discover biomarker candidates are updated frequently, especially with advanced high-throughput methodologies. Efficient text mining tools and algorithm development are extremely needed.

Many text mining technologies that are proposed by different groups, such as machine learning technologies including support vector machine [2], decision tree [3], Bayes classifier [4], and random forest [5], are used for text mining. Also the natural language processing technique is used to determine the structures and linguistic components of sentences and then parses the sentences in a bag of words, together with a statistic approach to get the matched results from the text databases. OMIM database [6] is one of the important databases for biomarker-related disease research. The MeSH Browser [7] is used to map disease associations to MeSH IDs. 

In this paper, we use a state machine to simulate the transforms of the biomarkers from individual entities to associated diseases and pathways as well as networks. Several abstracted templates are summarized from known expert experience and knowledge. The biomarkers are ranked based on the importance to the diseases and the citations of the literature from PubMed. Based on this template, every mined biomarker-related pathways, networks, and disease will be collected and matched with the templates. 

## 2. Method

All the biomarkers mentioned in this paper are mined from the PubMed database. For each biomarker candidate, we use a finite state machine (FSM) [8] to identify biomarker, pathways, and associated diseases. Only the candidates which are accepted by FSM are viewed as biomarkers. The association between the biomarkers and the diseases can be output to refine the biomarkers.

**Figure 1:**
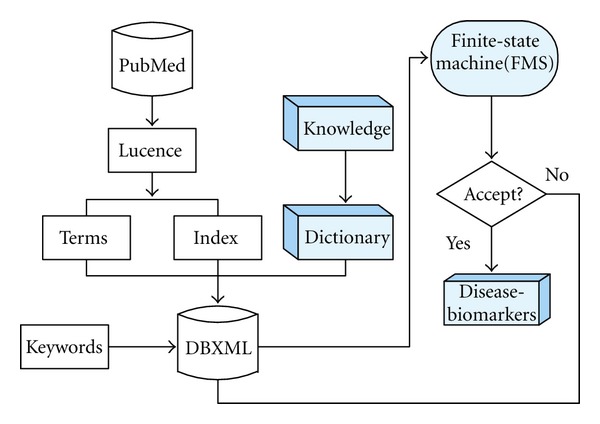
The flow chart of the biomarker discovery.

As shown in Figure 1. The first step is to create a biomarker dictionary, the second step is construct a DBXML [9] database, and the third step is using the finite state machine to conform the disease-related biomarkers. We first create our DBXML database from the PubMed database. The Lucence technique is used to split the document into a bag of words and extract terms such as gene names, interaction relationships, pathways, and network names. In the mean time, based on our domain knowledge, we construct the dictionary for further analysis. Based on the Lucence parsed terms and dictionary, the DBXML database is created for biomarker extraction. To retrieve the keyword from the DBXML database, exact matching, fuzzy matching, and list matching methods are used to match the terms saved in DBXML. If the end state of the FMS is in an acceptance state, the keywords-related genes, proteins, or small molecules are marked as biomarkers.

### 2.1. Construct a Database for Gene/Protein and Disease

We first construct a database which includes categories according to their names, diseases, interactions, pathways, and network information. Then, we collect a list of diseases, gene/protein and so on, and then put them into the dictionary. The structure of the dictionary is shown as Table 1.

We use a dynamic method to collect the full-text document, and then the Lucence is applied to split the word. For Lucence, we need to delete the old document and create new Lucence document index. The Lucence document contains three paths, content and the index of the document, the terms, and the modified date. 

Each word is separated by a series of phrases, and we use the dictionary to parse the full-text and then divide them into several primary categories: molecule names, interaction keywords, and verbs. After we extract the keywords, we construct the segment of the xml document for those keywords. The protein name is an entity, and the interaction represented the relation of the entity which is used to extract the relationships between diseases, genes, mutations, and proteins. We give an example of an xml segment extracted from PubMed as follows:

 <protein  id=010>
    <name>P53 </name>
    <interact>MDM2</interact>
 </protein>

If the words cannot match the dictionary, it will be ignored. Some keywords can be removed from the database as they are not suitable for our definition. Additional tags can also be added by the users. Table 1 shows the dictionary of the biomarkers.

Our database does not contain interaction pairs and pathways. We will dynamically parse online databases for the protein/gene names and build the interaction network.

### 2.2. Using the FSM to Identify Biomarkers

We used the finite state machine (FSM) to identify the biomarkers in our database. The FSM is a state machine which has a start node, accepting node, input entities, and relations. The roles contain the information of each entity such as genes, proteins, and small molecules. 

**Figure 2:**
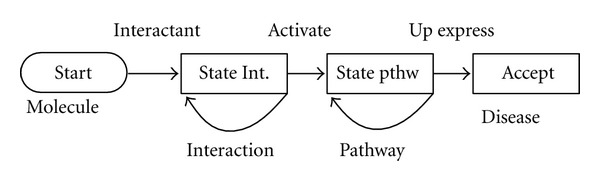
The identification of the biomarker using the finite state machine.

In this paper, the FSM for identifying biomarkers is regarded as a template which serves to match corresponding biomarkers as shown in Figure 2. In addition, the template can be modified by users. Our methods include exact matching, fuzzy matching and list matching. For disease, we use the exact match method, for all molecules, we use fuzzy matching, and for interaction, we use list-member matching.

For the list member of interactions, the list members are defined as ILIST(*P*_*a*_)∶ = (*P*_1_, *P*_2_, *P*_3_,…*P*_*n*_), where *P*_*a*_ interacts with *P*_1_, *P*_2_, … *P*_*n*_, which dynamically parse online databases. We construct the protein-protein interaction network around *P*_*a*_ in the FSM. We also obtain the pathway from the KEGG database. 

The FSM includes <left-context FSM>, <entity FSM>, and 〈right-context-FSM〉. The roles of the entities are determined by the context of the left and right neighbors of the entities.

For example, for the entity P53 which is a protein, we determine the role of the entity as follows:

 If < right-context>
    = <(*“*express*”“*present*”*)(*“*in*”“*at  pathway*”*)>
     Then  entity  role = in  the  pathway

The output of the FSM is the track nodes between <Disease> ⇔ <Potential Biomarker> which include paper name and author name. The FSM is shown in Figure 2.

## 3. Experimental Results

Based on our framework, a query on liver cancer and the candidate biomarkers are report as Table 2. 

**Figure 3:**
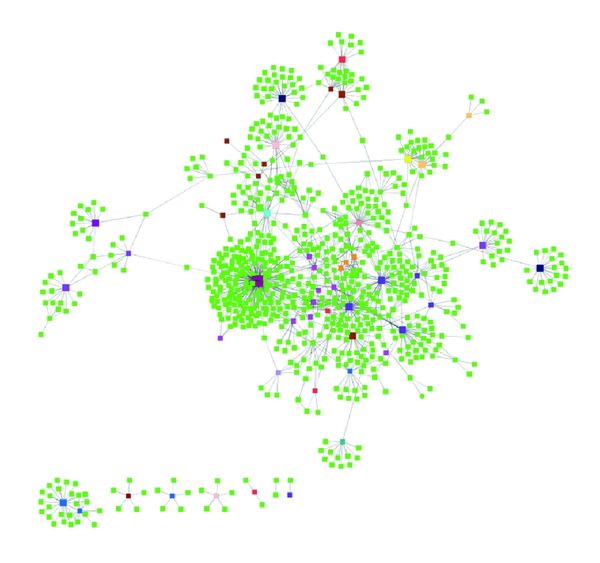
A diseases-related gene associated network. Green nodes are genes, and the nodes in other colors are diseases.

 In a query process, we dynamically parse the identified genes/proteins and construct the interact network. We then use Cytoscape software [10] to display the interaction network shown as Figure 3.

## 4. Conclusions

The proposed method is based on text mining technique from the PubMed database, combined with the full text search-engine technology (Lucence), a complex network of biological and signaling pathways. First, we construct a database based on a dictionary; second, we use a FSM to identify the biomarkers; finally, we output the disease-associated biomarkers. This research offers a comprehensive text mining to discover biomarkers.

## Figures and Tables

**Table 1:** The dictionary of the biomarkers.

Gene	Protein	Pathway	Disease
P53	P53	Ras	Diabetes
APC	APC	Wnt	Breast cancer
MDM2	Pten	Death receptor pathway	Liver cancer
Ras	HCC	Ether lipid metabolism	Huntington
Axin-1	HPR	Thiamine metabolism	Liver cirrhosis
	LCE2B	Porphyrin and chlorophyll	Prostate cancer
	AXIN1	Metabolism	Leukemia
	SLC22A1		

**Table 2:** The list of biomarker-disease associations mined from PubMed.

EntrezID	Gene name	Symbol
11914	ALPHA 1,4-GALACTOSYLTRANSFERASE	A4GALT
3558	ACETOACETYL-COA SYNTHETASE	AACS
5758	ABHYDROLASE DOMAIN CONTAINING 1	ABHD1
18925	ACYL-COA THIOESTERASE 12	ACOT12
18925	ACYL-COA THIOESTERASE 12	ACOT12
17809	ACYL-COA THIOESTERASE 2	ACOT2
17766	ACYL-COA THIOESTERASE 4	ACOT4
15426	ACYL-COA SYNTHETASE BUBBLEGUM FAMILY MEMBER 1	ACSBG1
11191	ACYL-COA SYNTHETASE BUBBLEGUM FAMILY MEMBER 2	ACSBG2
